# Heterogenous nanocomposite catalysts with rhenium nanostructures for the catalytic reduction of 4-nitrophenol

**DOI:** 10.1038/s41598-022-10237-5

**Published:** 2022-04-14

**Authors:** Piotr Cyganowski, Anna Dzimitrowicz

**Affiliations:** 1 Department of Process Engineering and Technology of Polymer and Carbon Materials, Faculty of Chemistry, Wroclaw University of Science and Technology, Wybrzeze S. Wyspianskiego 27, 50-370 Wroclaw, Poland; 2 Department of Analytical Chemistry and Chemical Metallurgy, Faculty of Chemistry, Wroclaw University of Science and Technology, Wybrzeze S. Wyspianskiego 27, 50-370 Wroclaw, Poland

**Keywords:** Pollution remediation, Nanoparticles, Materials for energy and catalysis

## Abstract

Stable and efficient heterogenous nanocatalysts for the reduction of 4-nitrophenol (4-NP) has attracted much attention in recent years. In this context, a unique and efficient in situ approach is used for the production of new polymeric nanocomposites (pNCs) containing rhenium nanostructures (ReNSs). These rare materials should facilitate the catalytic decomposition of 4-NP, in turn ensuring increased catalytic activity and stability. These nanomaterials were analyzed using Fourier-Transformation Infrared spectroscopy (FT-IR), transmission electron microscopy (TEM), and X-ray powder diffraction (XRD). The efficiency of the catalytic reaction was estimated based on the acquired UV–Vis spectra, which enabled the estimation of the catalytic activity using pseud-first order modelling. The applied method resulted in the successful production and efficient loading of ReNSs in the polymeric matrices. Amino functionalities played a primary role in the reduction process. Moreover, the functionality that is derived from 1.1′-carbonyl imidazole improved the availability of the ReNSs, which resulted in 90% conversion of 4-NP with a maximum rate constant of 0.29 min^−1^ over 11 subsequent catalytic cycles. This effect was observed despite the trace amount of Re in the pNCs (~ 5%), suggesting a synergistic effect between the polymeric base and the ReNSs-based catalyst.

## Introduction

Due to the unique properties of the –NO_2_ group, nitroaromatic compounds (NARs) serve as key building blocks for the manufacturing of fine chemical products. This makes NARs the largest group of chemicals used on an industrial scale^1–4^. Moreover, large quantities of NARs are detected on a daily basis in the exhaust of diesel engines. These significant waste sources render NARs as one of the major environmental and health hazards, as according to the International Agency for Research on Cancer (IARC), NARs significantly contribute to the risk of lung, bladder, and pancreas cancers, and in case of children also urinary track and neurological-related cancers^5,6^.

The most popular method for neutralizing NARs is the direct reduction of –NO_2_ to –NH_2_ groups. Such an approach is very convenient, as aromatic amines (AMMs) are crucial for the manufacturing of i.e. large-scale pharmaceuticals^7,8^. However, for this to occur, the reduction requires a catalyst, and as such the application of metallic nanostructures (NSs) as catalysts is particularly important^9^. NSs enable the carrying out of the effective reduction of NARs to AAMs under mild conditions. This has made the nanocatalysts (NCats) of NSs one of the most important scientific directions [9, 10, 49]. To date, the reduction of NARs was tested over various NCats, including AuNSs, AgNSs^10^, PtNSs, and PdNSs^11,12^. Based on this research, the PtNSs and PdNSs offer an extraordinary and unique activity, leading to the complete reduction of NARs—even at trace amounts of the NCats^11,12^. Therefore, it is expected that the barely known nanomaterial (NM): rhenium NSs (ReNSs) would significantly boost the catalytic activity of NCats towards the reduction of NARs. Literature and practice provide numerous high-tech applications of Re in areas such as the aerospace, nuclear and petrochemical industries. Metallic Re is indispensable in the catalytical processes related to increasing the octane number of commercial gasolines, as well as Fisher–Tropsh and ammonia syntheses^13–16^. The literature also proves that the NCats of ReNSs outperformed PGM catalysts in the decomposition of 4-nitroaniline^17^, nitrobenzene, 4-nitrophenol, 2-nitroaniline, 2,4-dinitrophenol, and 2,4,6-trinitrophenol^18^. In this context, in our previous works we have revealed a superior catalytic activity of homogenous^19^ and heterogenous^20^ NCats with ReNSs towards reduction of 4-nitrophenol and 4-nitroaniline. However, ReNSs are difficult to be obtained. The worldwide scientific literature provides only a few reports on the production of ReNSs. This includes the hot-injection synthesis of Co-ReNSs^21^, and also the production of ReNSs using pulsed-laser deposition^22^, electrodeposition^23^, gamma radiation^24^, and chemical vapour deposition^25^ approaches. Recently, we have proposed two new approaches for the synthesis of Re-based NMs. This included application of reaction discharge systems^19^ as well as the reduction-coupled adsorption onto amino functionalities of an anion-exchange resin^20,26^.

Based on the reviewed literature and gained experience, the following challenges related to the fabrication and application of ReNSs-based NCats for the reduction of NARs can be identified. First, it is possible to increase the rate and efficiency of the catalytic reduction of NARs by using ReNSs instead of other NSs. Second, ReNSs should be stable and possible to be re-used. Third, the synthesis of ReNSs could be facilitated. To address these challenges, a new approach is proposed in this work. This involves the synthesis and loading of ReNSs into polymeric anion exchange resins, in turn leading to the fabrication of new, heterogenous NCats with ReNSs without addition of any external reducing agent. The proposed method exploits the amino functionalities present on a polymer’s surface to make them serve as reducing and capping agents towards Re(VII) and resultant ReNSs, respectively. The unique in situ method facilitates the production and stabilization of ReNSs in the course of the reduction-coupled adsorption of the ReO_4_^−^ anion. Moreover, the morphology of the so-prepared polymeric nanocomposites (pNCs)—polymeric beads, makes them easy to use and facilities the recycling of ReNS-based NCats.

## Results and discussion

### Synthesis of the polymeric base with amino reactors

The base for the pNCs with ReNSs was synthesized by modifying the copolymer of vinylbenzyl chloride (VBC) and divinylbenzene (DVB) using amines, in turn revealing the reducing and capping properties. The success of this process was estimated by determining the Cl and N concentration before and after the modification, as well as by evaluating the spectra recorded using Attenuated Total Reflectance Fourier Transformation Infrared Spectroscopy (ATR-FTIR) displayed in Table 1 and Supplementary Fig. S1, respectively.

**Table 1:** Characteristics of the polymeric matrices for the pNCs with ReNSs.

Polymer	Amine	W^a^	Cl(1)^b^	Cl(1)^c^	N^d^
VBC-co-DVB	–	–	4.62 × 0.09	–	–
VBC-co-DVB (modified)	HEP	0.90 × 0.05	0.21 × 0.09	2.74 × 0.12	6.64 × 0.28
	BAPP	0.60 × 0.08	0.13 × 0.10	2.62 × 0.21	7.32 × 0.25
	CIM	1.03 × 0.11	0.16 × 0.14	1.72 × 0.15	6.58 × 0.31

^a^H_2_O regain [g g^−1^]

^b^Covalently-bonded Cl [mmol g^−1^]

^c^Ionic chlorine [mmol g^−1^]

^d^N concentration [mmol g^−1^].

**Figure 1:**
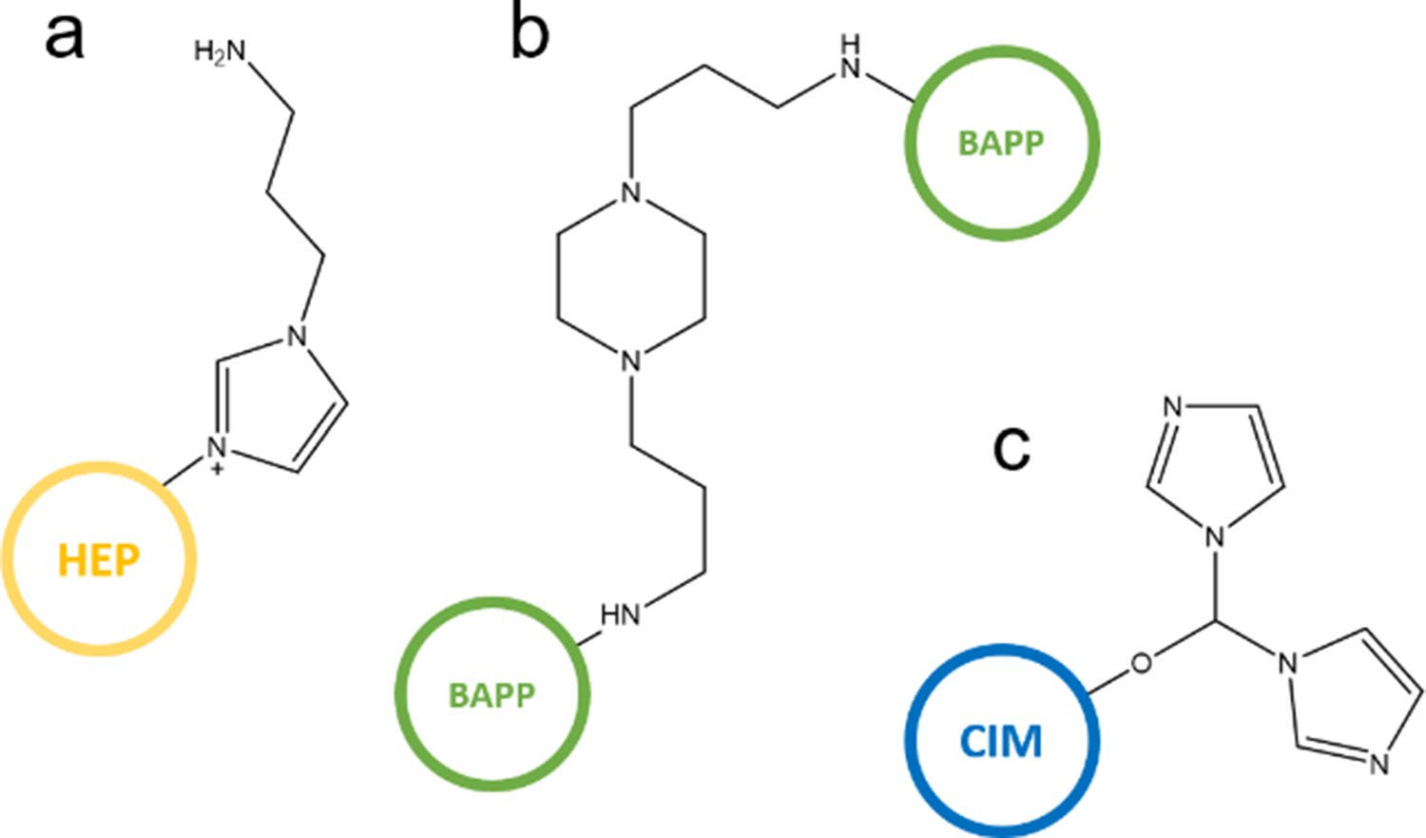
Simplified scheme of the structure of the amino functionalities in the (**a**) HEP, (**b**) BAPP and (**c**) CIM.

As can be seen in Table 1, the initial Cl(1) concentration in the VBC-co-DVB copolymer (4.25 mmol g^−1^) decreased by approximately 97% as the result of modification. This result is consistent with Supplementary Fig. S1, where bands at 704 cm^−1^, indicating C–Cl stretching in the –CH_2_Cl groups, faded when compared to the corresponding bands recorded for the HEP, BAPP and CIM samples. Simultaneously, a band at 1264 cm^−1^ (VBC-co-DVB), indicating C–H deformations with the attached Cl, disappeared completely as the result of modification^27^. These observations confirm that the Cl in the –CH_2_Cl groups was indeed substituted. After modification, all the samples revealed an N content (N, Table 1), up to 30% of which was able to be protonated in HCl (based on Cl(2), Table 1). This suggests that the amines were successfully introduced into the VBC-co-DVB copolymer. Furthermore, evidence of this can be found in the form of a set of new wide bands attributed to the N–H stretching observed for the 1-(2-hydroxyethyl)piperazine (HEP), 1,4-bis(3-aminopropyl)piperazine (BAPP), and 1,1′-Carbonyldiimidazole (CIM) at 3323, 3364, and 3393 cm^−1^, respectively. The N–H deformations in the –NH– groups were detected in the BAPP sample at 1263 cm^−1^. Additionally, C–N stretching in the –NH_2_ was also detected in the HEP at 1161, 1121 and 1082 cm^−1^^27^, however, this band was not visible in the case of the BAPP, which suggests that the latter one was attached to the VBC-co-DVB copolymer via*. –*NH_2_ groups. This conclusion is supported by the smallest water uptake (W, Table 1) of the BAPP, which suggests secondary crosslinking through –NH_2_ bridges. N-containing rings (aromatic and heterocyclic) are reflected by the bands at 1540, 1538, and 1537 cm^−1^ in the HEP, BAPP and CIM samples, respectively. Moreover, the appearance of bands at 1653 and 1659 cm^−1^ was attributed to the N=C–N stretching deformations, and the bands at 1357, and 1358 cm^−1^ were attributed to the asymmetric N–C–N stretching found in the case of the HEP and CIM, respectively^27^. The CIM sample also displays a fingerprint of C=O groups at 1685 cm^−1^, however, unexpectedly, the intensity of this band is weak instead of very strong. This, linked with the bands at 1016 and 898 cm^−1^, which can be assigned to the C–O–C^27^ vibrations, suggest the primary role of the carbonyl group in the modification of VBC-co-DVB. Based on these results, it is possible to propose the structure of functionalities present on the surface of anion exchange resins. These are displayed in Fig. 1.

### Morphology of the pNCs with ReNSs

**Figure 2:**
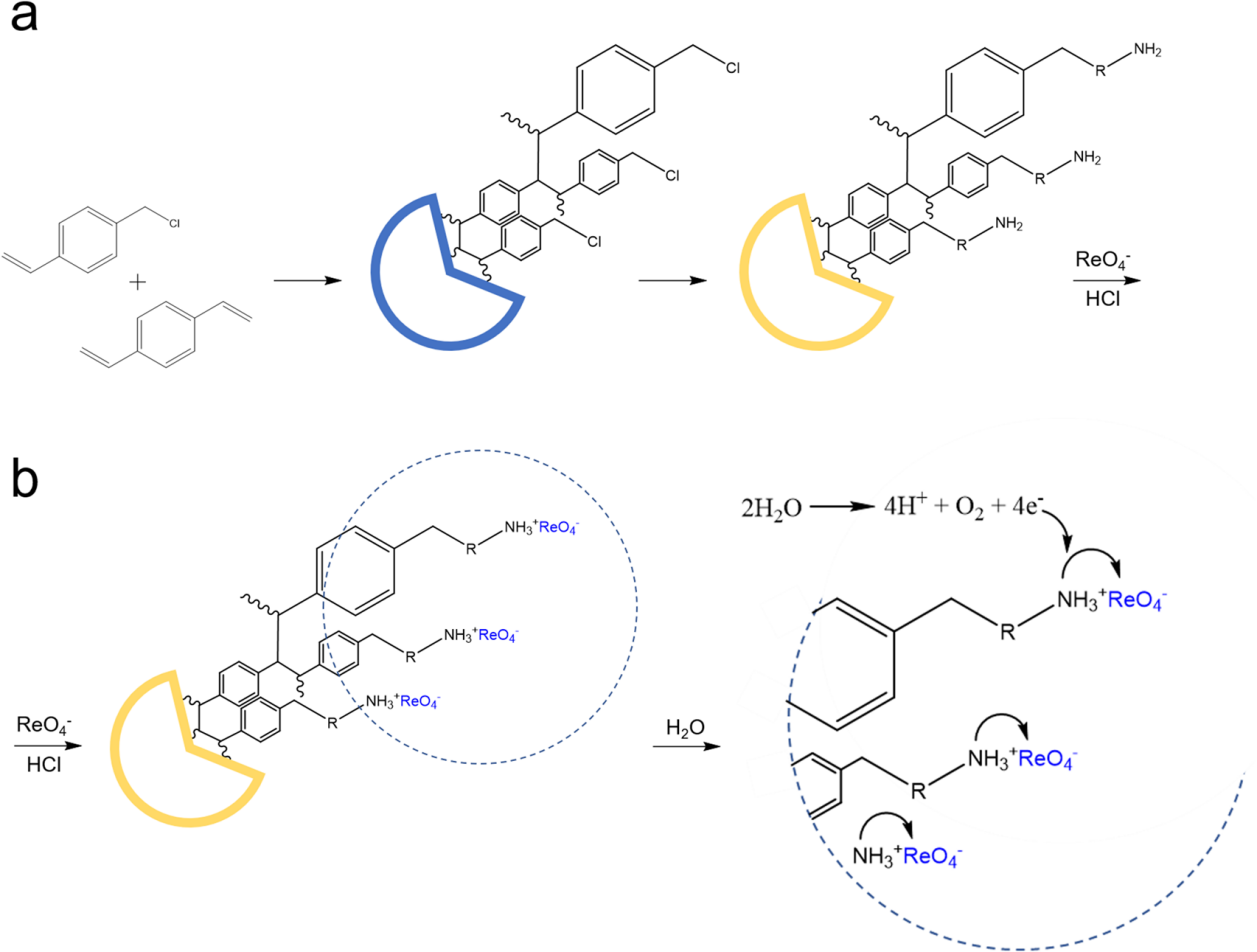
Synthesis of the pNCs with ReNSs. (**a**) Synthesis of the polymeric matrix, (**b**) Re(VII) loading and reduction.

As a result of the synthesis (see Fig. 2a) followed by adsorption coupled adsorption of the ReO_4_^−^ anion (see Fig. 2b), the ReNSs were successfully loaded and stabilized into the amino-functionalized VBC-co-DVB copolymers.

**Figure 3:**
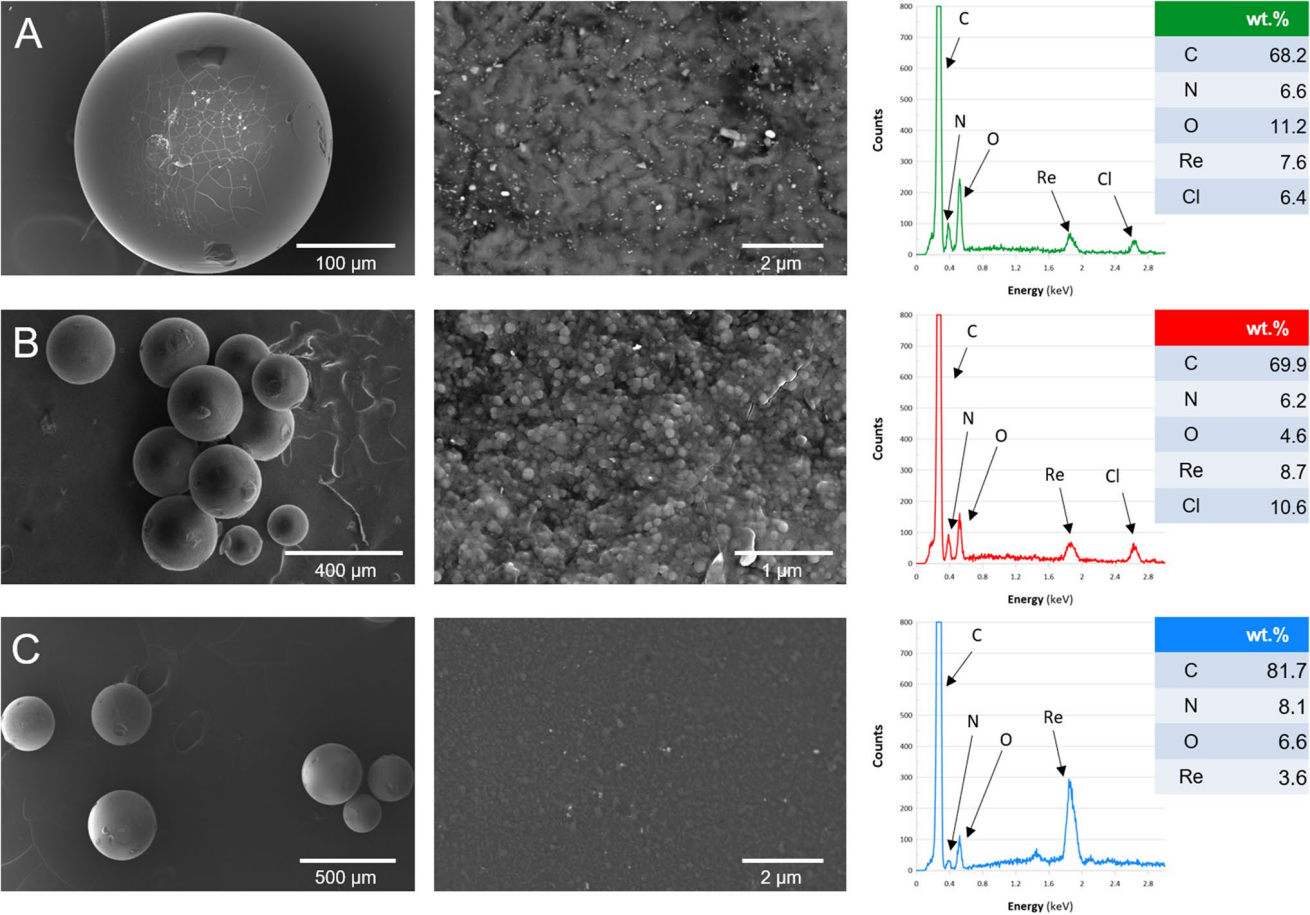
SEM photomicrographs and EDX spectra of (**A**) Re@HEP, (**B**) Re@BAPP, and (**C**) Re@CIM samples. The elemental composition is an average value obtained from 3 EDX micro elemental analyses.

To evaluate the morphology of the resultant ReNSs Scanning Electron Microscopy (SEM), Transmission Electron Microscopy (TEM), and High Resolution TEM (HRTEM) analyses were carried out. Figure 3 displays SEM photomicrographs and EDX spectra of the analysed samples. As can be seen, the suspension VBC-co-DVB copolymers (polymeric base) indeed revealed spherical morphology, that size ranged from 100 to 500 μm (Fig. 3, first column). Closer look at the surface of the Re-loaded samples (Fig. 3, second column) reveals various structures characterized by observable phase contrast. Based on the acquired EDX spectra (Fig. 3, third column), these were recognized as ReNSs. Besides Re, the analysis of elemental composition revealed the presence of C (originating from polymeric matrix), N (originating from amino-functionalities), O (originating from ReO_4_^−^ and/or amino-functionalities), and Cl (originating from residual ‒CH_2_Cl groups in the polymeric matrix). The size of the largest ReNSs did not exceed 100 nm. Simultaneously, it was expected, that the ReNSs observed on the surface of the samples would be larger, as compared to those occluded in the polymers’ grain. It was found in our previous works^28–30^, that the reduction-coupled adsorption of Au, Pt and Pd chlorocomplexes was controlled by the concentration gradient at solid–liquid interface. Thus, larger NSs were observed on the surface of polymeric nanocomposites, while much smaller NSs were found in the inner part of polymeric spheres^28–30^. Therefore, it was expected, that similar phenomenon could be observed in the case of ReO_4_^−^ reduction coupled adsorption as well. At this point, it must be also mentioned, that the SEM photomicrographs (Fig. 3) suggested, that the samples Re@HEP (Fig. 3A) and Re@CIM (Fig. 3C) contained much more ReNSs on their surface as compared to the sample Re@BAPP (Fig. 3B). Based on our previous studies on the production of AuNSs^28–30^, the process of reduction on amino functionalities produces more NSs in the presence of free ‒NH_2_ groups and additional chelating atoms besides N. As such, the samples HEP and CIM offered such a structure while the sample BAPP did not (see Fig. 1 for details).

**Figure 4:**
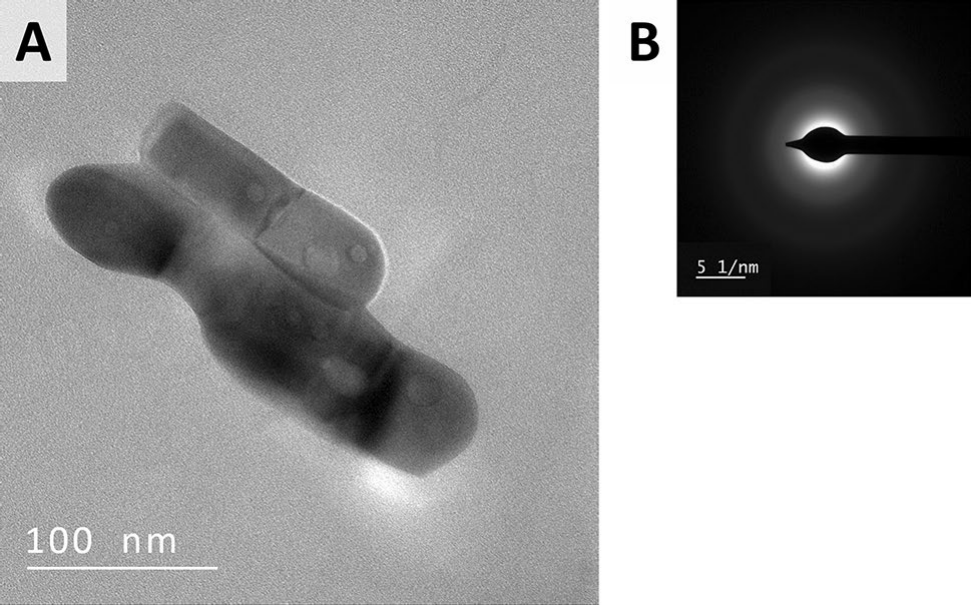
(**A**) TEM photomicrograph and (**B**) SAED pattern of the ReNSs found in the slices of Re@BAPP.

**Figure 5:**
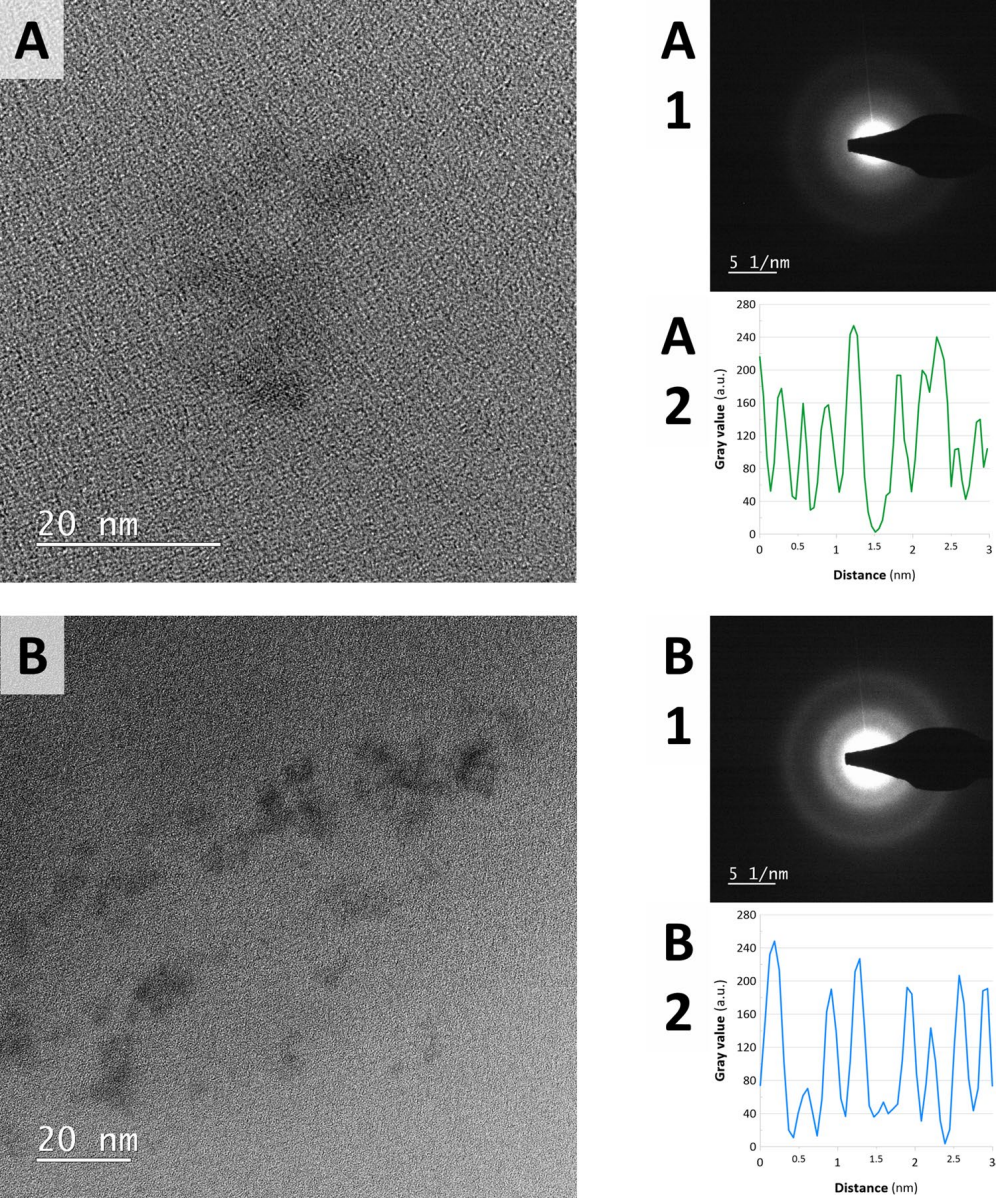
HRTEM photomicrographs, SAED patterns, and d-spacings profile of (**A**,**A1**,**A2**) Re@HEP, and (**B**,**B1**,**B2**) Re@CIM, The profiles displayed in panels (**A2**) and (**B2**) were acquired on the basis of a contrast difference in the observed planes of nanoparticles.

Because it was expected that the synthesized samples contained much smaller ReNSs than these revealed by the SEM analysis further TEM and HRTEM, analyses were carried out on the cross-sections of the polymers. Figure 4 displays TEM photomicrograph of Re@BAPP sample. In turn, Fig. 5 shows HRTEM photomicrographs of Re@HEP and Re@CIM samples, that contained NSs much smaller that these observed in Re@BAPP.

Based on the analyses, the synthetic route (see Fig. 2 for details) resulted in the loading of NSs into the polymeric matrices. Moreover, the Energy Dispersive X-Ray (EDX) spectra provided in Fig. 3, and Supplementary Fig. S2 indicate that these are indeed Re-based NSs. There are only a few reports in the literature that focus on Re-based nanomaterials^31,32^. Based on these reports, Re can form either Re-oxide or Re^0^ NSs, the identification of which is challenging. It has been noticed, however, that the NSs of Re-oxides should reveal characteristic reflections in both Selected Area Energy Diffraction (SAED) and X-Ray Powder Diffraction (XRD) patterns^33–35^. Meanwhile, the SAED patterns displayed in Figs. 4 and 5, as well as XRD spectra displayed in Supplementary Fig. S3 suggest an amorphous structure of the tested NSs. This was previously recognized as an effect attributed to the presence of Re–Re bonds without oxide-doping^33–35^, which tend to shift both SAED and XRD spectrums into the amorphous region. This suggests the formation of ReNSs with Re at a 0 oxidation state. However, as displayed in Fig. 5A,B (HRTEM photomicrographs) it is possible to notice clear, and regular contrast between atom planes within analysed ReNSs. Simultaneously, Supplementary Fig. S4 displays processed photomicrographs with the highlighted structures used for the further discussion (full resolution HRTEM photomicrographs are accessible from the corresponding author on request). This suggests, that although the SAED and XRD spectra reveal amorphous structure of the nanoparticles, they are not entirely so. Based on the grayscale profiles displayed in Fig. 5A2 and B2 it was possible to determine a set of d-spacings between the observed planes. These allowed to identify species of Re that contribute to the ReNSs. In the case of Re@HEP sample, d-spacings allowed to determine the presence of Re_3_O_10_ (d-spacing 0.337 nm)^32^, ReO_2_ (d-spacing 0.283 nm)^32,36^, and Re_2_O_7_ (d-spacing 0.190 nm)^32^ species. Also, consistently with SAED and XRD patterns, the Re(0) was also found (d-spacing 0.223 nm)^36,37^. In the case of Re@CIM sample, d-spacings suggested the presence of ReO_3_ (d-spacing 0.378 nm)^32^, Re_3_O_10_ (d-spacing 0.341 nm)^32^, ReO_2_ (d-spacing 0.291 nm)^32,36^. In this case, Re(0) was also found (d-spacing 0.225 nm)^36,37^ as suggested by SAED and XRD patterns.

Based on these observations, it can be concluded that the ReNSs formed in the Re@HEP and Re@CIM samples were the blend of different Re species with Re at 0, IV, VI and VII oxidation states. Simultaneously, no similar observations has been made in the case of Re@BAPP sample. This does not exclude formation of the similar blend, as the NSs displayed in Fig. 4A are much bigger as compared to these displayed in Fig. 5, and thus, observing atomic planes might have been impossible. At this point it must be also mentioned, that besides the size difference, the ReNSs found in the sample Re@BAPP were much more dispersed and were not so frequently found as in the case of Re@HEP and Re@BAPP sample. This observation is consisted with the SEM analysis and suggests that the BAPP matrix could not had been suitable for the production of nanocomposites with ReNSs. The size of the ReNSs ranged from 2–50, 50–150, and 1–70 nm in the case of the Re@HEP, Re@BAPP and Re@CIM, respectively. Because these sizes of ReNSs in Re@HEP and Re@CIM are similar, it is hypothesized that the resultant catalytic activity will be somehow correlated with the Re concentration in a sample, and this in turn should be proportional to the amount of amino reactors (N concentration, Table 1)^28^. To verify this, the pNCs with ReNSs were digested in concentrated HNO_3_ and analysed using Flame Atomic Absorption Spectroscopy (FAAS). This allowed the concentration of Re in each pNC sample to be determined, as displayed in Table 2.

**Table 2:** Re concentration in pNCs: Re@HEP, Re@BAPP and Re@CIM.

Sample	Concentration* of Re (%)
Re@HEP	10.54 × 0.51
Re@BAPP	8.93 × 1.02
Re@CIM	5.25 × 0.74

*Expressed as g of Re per g of the polymer.

According to the obtained results, it can be stated that the Re concentration is indirectly linked with the N concentration (Table 1), i.e. it seems that a direct dependence is found only for protonated amines (–NH_3_^+^Cl^−^), the concentration of which can be estimated by ionic chlorine, as seen in in Table 1 (Cl(2)). In our previous works, there was a clear proportional dependence between the concentration of N and Au^0^^28,29^. However, within the present studies, not all of the N atoms in the HEP, BAPP and CIM were able to protonate (and thus to perform anion exchange), it can therefore be estimated that 41% of the N in the HEP, 34% of the N in the BAPP, and 24% of the N in the CIM was able to perform reduction-coupled adsorption of ReO_4_^−^. As a result, both the active amino reactors and the Re concentration can be placed in the following order: Re@HEP > Re@BAPP > Re@CIM.

### Catalytic activity

**Figure 6:**
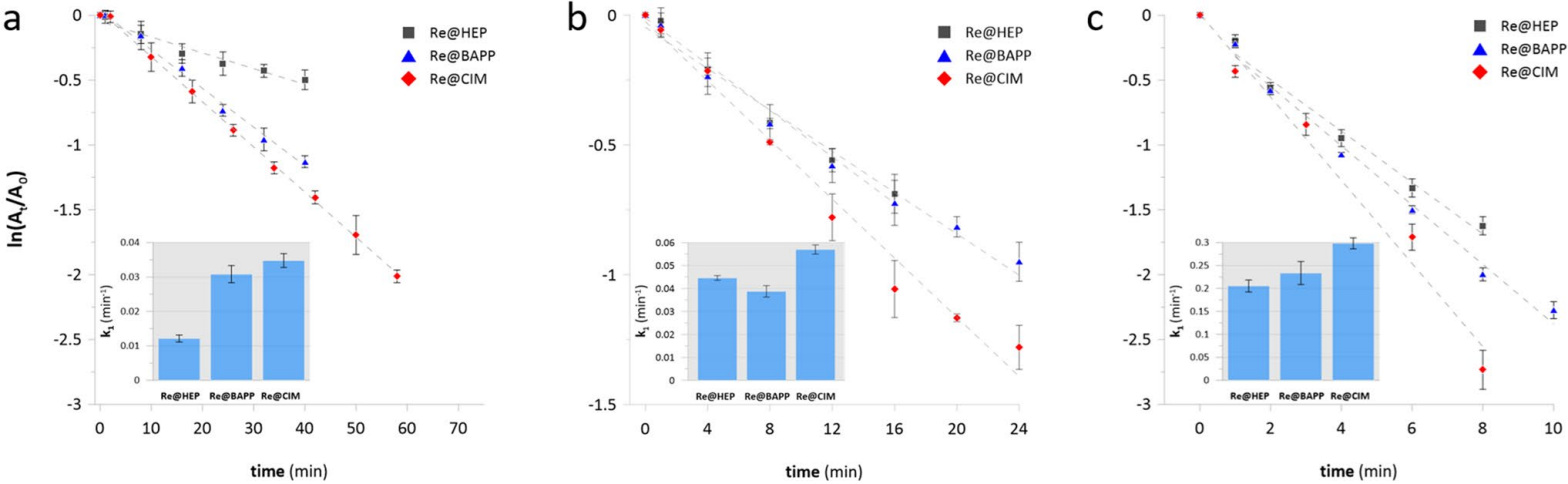
4-NP reduction first-order kinetics plots. The graphs represent processes using Re@HEP, Re@BAPP and Re@CIM NCats: (**a**) 5 mg, (**b**) 10 mg, (**c**) 50 mg.

Because the synthesis and loading of the ReNSs in the pNCs was successful, the Re@HEP, Re@BAPP and Re@CIM samples were used as heterogenous NCats for the reduction of 4-nitrophenol (4-NP). First, the mass of a catalyst was optimized towards the reduction of 4-NP. The catalytic reaction was carried out for 5, 10, and 50 mg of each NCat. The recorded spectra, with characteristics bands at 295, 318 and 400 nm, are displayed in Supplementary Fig. S5. The values of absorbance at 400 nm were then re-calculated to obtain the *lnA*_*t*_*/A*_*0*_ vs.* t* plots and rate constants (*k*_*1*_) for each NCat mass. These are displayed in Fig. 6.

**Figure 7:**
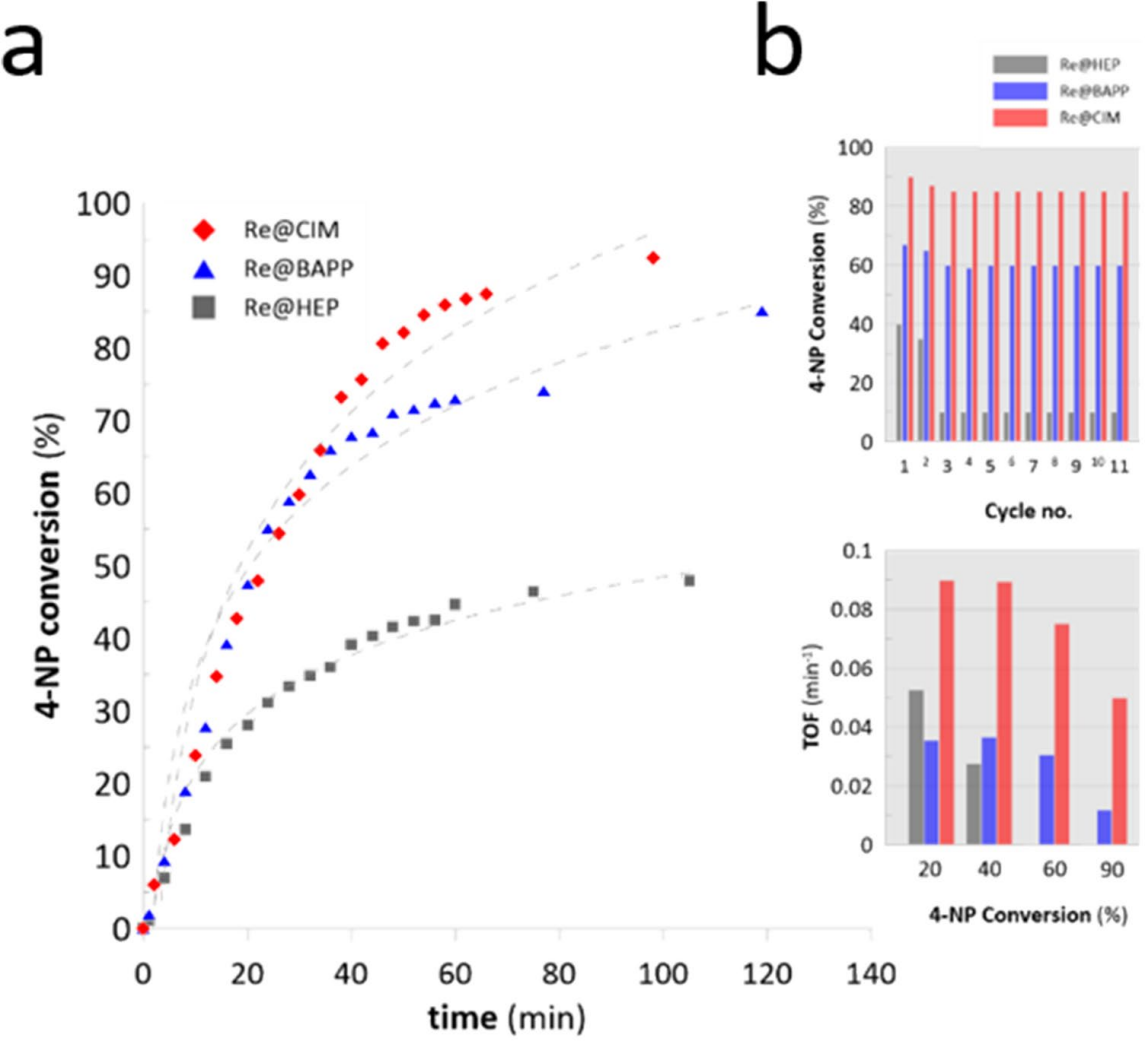
(**a**) 4-NP conversion over time in the first catalytic cycle, (**b**) 4-NP conversion over 11 catalytic cycles, and the TOFs achieved using 5 mg of NCats in the 1st catalytic cycle.

Based on the results displayed in Fig. 6 and Supplementary Fig. S5, it can be concluded that the most efficient NCat was Re@CIM. 5 mg of the sample hydrogenated 90% of the 4-NP within 58 min, while 50 mg of this sample reduced 90% of the 4-NP within 8 min. These results are better than when compared to those revealed by the Re@BAPP NCat; the apparent rate constants (*k*_*1*_) of the Re@CIM were 35–20% greater. The smallest values of *k*_*1*_ combined with the smallest 4-NP conversions were observed in the case of the Re@HEP. In this case, the *k*_*1*_ values were up to 50% smaller when compared to the Re@CIM. These results seem to be inconsistent with the Re concentration given in Table 2. Despite the fact that the Re@CIM sample reveals the smallest Re content (~ 5%), its catalytic activity is greater when compared to the Re@HEP (~ 11% Re), and similar when compared to the Re@BAPP (~ 9% Re). This suggests that there is a synergistic effect between the polymeric matrix and the ReNSs. It has previously been recognized that the ReO_4_^−^ oxoanion prefers amino functionalities of a “cage-like” structure within which it could fit^15,38,39^. As such, CIM functionality (see Fig. 1) offers such a structure. It can therefore be concluded that the polymeric matrix of the Re@CIM made the ReNSs more accessible, resulting in the better catalytic performance. Yet another support for this conclusion can be found in Supplementary Fig. S5 when comparing the UV/Vis spectra of the catalytic reactions performed using 5 mg of NCats. Despite having the smallest Re concentration, the Re@CIM enabled almost the entire decomposition of the 4-NP, while the maximum conversions achieved with the aid of Re@HEP and Re@BAPP were 40 and 87%, respectively, even though they contained more Re (see Table 2). The data presented in Fig. 6 were calculated as a function of time (*t*), defined as the time before which the catalytic activity started to fade and fall out of the kinetic model. However, the catalytic reactions were carried out until the maximum 4-NP conversions were achieved (as displayed in Supplementary Fig. S5). This enabled further evaluation of the kinetic behaviour when applying 5 mg of NCats. All of the acquired data were re-calculated to Turnover Frequencies (TOF), which are a measure of the number of catalytic reactions carried out on a single catalytic centre over time^40^. The 4-NP conversion vs*.* time plots, as well as the values of the TOFs for 20, 40, 60, and 90% 4-NP conversions are displayed in Fig. 7a.

The calculated time-dependent 4-NP conversions and TOF values are consistent with the conclusions stated above. The Re@CIM reveal the greatest values of TOF of 0.09, and 0.05 min^−1^ for 20 and 90% of 4-NP conversion, respectively. These values were at least twice as high as the corresponding values calculated for the Re@CIM and Re@BAPP. Moreover, the TOFs calculated for the Re@CIM only slightly decrease over time up to 60% of the 4-NP conversion. At 90% of the 4-NP conversion, the TOF value of the Re@CIM is still 5-times higher when compared to the Re@BAPP (0.05 vs*.* 0.01 min^−1^), while conversions of 60 and 90% were impossible using Re@HEP despite its twofold greater Re concentration when compared to the Re@CIM. This suggests a greater availability of ReNSs in the Re@CIM when compared to other NCats, despite the decreasing concentration gradient as the reaction proceeds. This in turn indicates the primary role of the polymeric matrix in facilitating the use of ReNSs-based NCats.

It can be stated that Re@CIM is very efficient. Applying 5 mg of this NCat yields 2.65 × 10^–6^ mol ReNSs. The use of this trace amount of NCat was facilitated by the polymeric matrix, which ensured its stability and accessibility. As a result, the Re@CIM remained catalytically active over the process, yielding high 4-NP conversions. However, yet another advantage of the synthesized NCats is their morphology, which facilitates their separation and re-use. Therefore, the NCats (5 mg) were used in the reduction of the 4-NP during the 11 subsequent cycles. The reaction times were taken as defined for achieving the maximum 4-NP conversions in the 1st catalytic cycle, i.e*.* 40, 40, and 58 min for the Re@HEP, Re@BAPP and Re@CIM, respectively. After completion of each reaction run, the NCat was simply separated by filtration and directly introduced into yet another cycle. As can be seen in Fig. 7b, the Re@CIM only slightly loses its activity. In this case, 4-NP conversion drops from 90 to 85% over 3 cycles, and remains at 85% until the last catalytic reaction. A similar observation was made in the case of the Re@BAPP (67 to 60% drop of 4-NP conversion). However, it can also be noticed that the Re@HEP sample loses 75% of its catalytic activity after the 2nd reaction run. This seems to support the conclusion that other NCats are characterized by a facilitated availability of ReNSs.

The scientific literature provides only a few of examples, where Re-based NCats were used for the catalytic reduction of 4-NP. These are summarized in Table 3.

**Table 3:** Efficiency of Re-loaded pNCs in the catalytic reduction of NARs.

Catalyst	NAR^a^	k_1_^b^	Comment	References
Re-nanocluster	4-NP	0.06	Homogenous catalyst	^41^
O-doped raw ReNSs	4-NP	0.160	Homogenous catalyst obtained in reaction-discharge systems	^19^
ReNSs loaded onto carbon NSs	4-NP	8.99	Homogenous catalyst with well-developed standard surface area	
	NB	8.89		
	2-NA	8.72		
	4-NA	8.83		
	2,4-DNP	8.81		
	2,4,6-TNP	8.65		^18^
ReNSs with Re^0^ on anion exchange resin with 1,1′-Carbonyldiimidazole	4-NP	0.282	Limited stability, invevitable conversion to O-doped ReNSs	^20^
	4-NA	0.329		
O-doped ReNSs on anion exchange resin			High stability	
1-(2-hydroxyethyl)piperazine	4-NP	0.205		This work
1,4-bis(3-aminopropyl)piperazine		0.234		
1,1′-Carbonyldiimidazole[		0.293		

^a^Nitroaromatic compound; 4-NP: 4-nitrophenol; NB: nitrobenzene; 2-NA: 2-nitroaniline; 4-NA: 4-nitroaniline; 2,4-DNP: 2,4-dinitrophenol; 2,4,6-trinitrophenol.

^b^Pseudo-first order rate constant (min^−1^).

To the best of our knowledge there is one example of heterogenous NCat with ReNSs used for the catalytic reduction of 4-NP, and it is our previous work^20^. In that paper, we have prepared Re-loaded suspension copolymer with a functionality derived from CIM. The difference was, that the methodology led to the synthesis of ReNSs with Re^0^, by applying external reducing agent and eliminating oxygen from the environment in which the reduction of ReO_4_^−^ was carried out. The obtained *k*_*1*_ values of 4-NP reduction was 0.282 min^−1^ for 50 mg of the catalysts^20^, while in the present studies the corresponding *k*_*1*_ values were 0.205, 0.234, and 0.293 min^−1^ for Re@HEP, Re@BAPP and Re@CIM, respectively. Based on the ReNSs concentration (see Table 2 for details), and NCats mass applied (50 mg), the NCat with Re^0^ (~ 1% of ReNSs^20^) seems to be more efficient that the NCats obtained in the present studies^20^. This suggests that obtaining pNC with Re^0^ should be favoured as their catalytic activity is greater as compared to these achieved by O‒doped ReNSs presented here. However, based on previous results^20^ there is one serious limitation. pNC with Re^0^ raised problems with limited reusability of the so-prepared NCat^20^. After 2 catalytic runs, the Re^0^ converted into different O-doped ReNSs^20^. This resulted in the instant drop of the catalytic activity. Based on the literature review^18^, ReNSs are susceptible to interact with oxygen, hence is no point of preparing NCats with Re^0^ as they will react with oxygen anyway. The literature provides also examples of homogenous NCat with ReNSs. This includes *i.a.* ReNSs immobilized on carbon NSs with greatly developed porosity^18^. Such catalyst revealed *k*_*1*_ value of 8.99 min^−1^ during catalytic reduction of 4-NP. This was much greater than the greatest *k*_*1*_ value obtained in the present studies (0.282 min^−1^ for Re@CIM). However, it must be mentioned, that the heterogenous NCats proposed here offer reusability unachievable for the homogenous ones. On the contrary, the catalytic activity of pNCs obtained in the present work was greater as compared to raw-ReNSs obtained with the aid of cold atmospheric pressure plasmas^19^ used as homogenous catalyst for the reduction of 4-NP (*k*_*1*_ = 0.160 min^−1^).

## Conclusion

Within the present studies we successfully obtained a series of pNCs with ReNSs, in turn revealing an enhanced catalytic activity towards the reduction of 4-NP. The synthetic procedure involved obtaining anion exchange resins with amino functionalities of a heterocyclic or aromatic character, followed by the reduction coupled adsorption of ReO_4_^−^ oxoanion. The so-obtained NCats were effective in the catalytic reaction, leading to up to 90% of 4-NP hydrogenation. Despite its significantly lower Re concentration in the Re@CIM sample, it revealed a significantly greater catalytic activity when compared to other nanomaterials. Such an effect was achieved due to the synergistic effect between the polymeric matrix and the ReNSs. As a result, the “cage-like-structure” 1,1′-carboimidazole ligands present in the Re@CIM made the ReNSs more accessible and stable. This led to high reaction rate constants and high TOF values, as well as catalytic activity over the subsequent 11 cycles.

The scientific literature provides only some information regarding Re-based nanomaterials for the reduction of nitroaromatic compounds. The obtained NCats were more efficient than the Re-nanocluster homogenous catalyst, which decomposed 4-NP with *k*_*1*_ = 0.06 min^−1^^41^. What is more, the above-mentioned research is related to the homogenous catalyst. In this context, besides a higher catalytic activity, the solution proposed in the present studies offer all the perks that heterogenous catalysts bring (e.g. ease of use, stability and facilitated reusability). It must also be stated that there is yet another example of ReNSs that are stabilized on carbon nanostructures^18^. This type of homogenous NCat led to 4-NP reduction with *k*_*1*_ = 8.99 min^−1^. However, this case is also a homogenous catalyst that is supported on the carbon nanostructures, which are characterized by a well-developed standard surface area. On the contrary, the heterogenous NCat with Re^0^^20^ was no better than O‒doped ReNSs presented here. Although the catalytic activity of these two types of NCats was almost the same, the pNCs with ReNSs reported in the present work offered stability and reusability.

The developed heterogenous NCats with ReNSs were also more efficient when compared to both the Ru nanoclusters immobilized on activated carbon (*k*_*1*_ = 0.198 min^−1^)^42^ and the amino-modified AuNSs (*k*_*1*_ = 0.3 min^−1^)^43^. The obtained results are also a significant advancement when compared to our previous work^28^, where the AuNSs-loaded HEP-functionalized VBC-co-DVB copolymer reduced 4-NP with *k*_*1*_ = 0.071 min^−1^. The main advantage was that the above-mentioned heterogenous catalyst contained almost 40% of Au^0^, and was literally covered with golden dust. In the present studies, the rate constants of 0.035–0.28 min^−1^ were achieved for much smaller Re concentrations (~ 5%).

The applied synthetic protocol enables the synthesis of pNCs with ReNSs. These unique types of nanomaterials provide enhanced catalytic activity, exceeding the performances of other solutions. At the same time, the synergistic effect between the ReNSs and amino-functionalized polymeric matrix ensures outstanding stability, improved availability and an enhanced catalytic activity of the ReNSs it contains. This in turn could be exploited in the synthesis of a new generation of NCats, which is based on rare NSs other than ReNSs. These NSs could also be characterized by improved usability and stability, as well as increased catalytic activity.

## Experimental procedure

### Materials

The reagents for the synthesis of the polymeric base, including monomers: vinylbenzyl chloride (VBC, mixture of *m* and *p* isomers, 99%), divinylbenzene (DVB, 80%), free-radical initiator: benzoyl peroxide (BPO, pure), and amines: 1-(2-hydroxyethyl)piperazine (HEP, 97%), 1,4-bis(3-aminopropyl)piperazine (BAPP, 99%), and 1,1′-Carbonyldiimidazole (CIM, 97%), were acquired from MERCK (Poland branch). The monomers were purified using vacuum distillation, while the amines were used as received. The precursor for the ReNSs—ammonium perrhenate (NH_4_ReO_4_, 99%)—was acquired from Sigma-Aldrich Chemical Co. (branch Poland) and dissolved in 0.1 mol L^−1^ HCl in order to receive a stock solution with 1000 mg Re L^−1^. 4-nitrophenol (4-NP, 99%), which was used for the evaluation of catalytic activity, was purchased in MERCK (Poland branch). All other reagents, which are not named in this section, were purchased in Avantor Performance Materials Ltd. (Gliwice, Poland) and used as received. For all the tests reverse-osmosis (RO) water was used.

### Analyses and instrumentation

The Cl concentration (both covalent and ionic) in the polymers was determined using Schrödinger’s method^44^. The concentration of amino functional groups was estimated by determining the N concentration using Kjeldahl’s method^45^. To estimate the water regain of the polymer samples, wet polymers were weighted and dried. Then, the water content (g g^−1^) was calculated using mass balance. All the data with the provided SD values were acquired from 3 repetitions of the test.

The syntheses were evaluated by Attenuated Total Reflectance Fourier Transformation Infrared Spectroscopy (ATR FT-IR) using the JASCO FT-IR 4700 instrument (MD, USA). The spectra were recorded within the range of 4000 to 400 cm^−1^ with a resolution of 4 cm^−1^ and by applying 64 scans. The Re concentration was determined using Flame Atomic Absorption Spectroscopy (FAAS) using the GBC Avanta spectrometer (VIC, Australia). The analysis was performed after mineralization of the Re-loaded pNCs in concentrated HNO_3_ using the Büchi K-425 digester (Büchi Poland, Warsaw). The morphology of the pNCs with ReNSs was analysed using Scanning Electron Microscopy (SEM) and Transmission Electron Microscopy (TEM). The SEM analysis was performed using the FEI HELIOS NANOLAB 450HP instrument equipped with an Energy Dispersive X-Ray Analyser (EDX). The TEM and High-resolution TEM (HRTEM) analyses were performed for ultra-thin samples of the pNCs located on Cu grids using the FEI Tecnai G^2^ X-TWIN or FEI TITAN^3^ equipped instruments, respectively equipped with the EDX and the Selected Area Energy Diffractometer (SAED). The distance between planes of atoms (d-spacings) were calculated based on the HRTEM photomicrographs using ImageJ software in a following way. The HRTEM photomicrograph in its full resolution was profiled along a selected nanoparticle to determine a plot profile. This enabled determining a function of the distance (nm) between bright and dark planes determining d-spacings. The catalytic reaction was carried out using UV–Vis spectrophotometry with the aid of the Jasco V-530 (MD, USA) instrument.

### Synthesis of the ReNS-loaded pNCs

The ReNSs were introduced into the polymeric matrices using a unique in situ method involving the reduction-coupled adsorption of Re(VII) on amino functionalities.

The synthetic procedure is displayed in Fig. 2. First, the polymeric base was obtained via*.* copolymerization of VBC and DVB to receive a VBC-co-DVB copolymer in accordance with the method reported in the reference cited under no.^38^. The process was performed in a following way. First, the organic phase was prepared. This included setting the mixture of the functional monomer (VBC), crosslinker (DVB, 2 mol% in respect to VBC), toluene (50 wt.% in respect to VBC and DVB), and free-radical initiator (BPO, 2 mol% in respect to VBC and DVB). Second, the water phase was prepared. To reach this aim, RO water was introduced into glass tubular reactor equipped with a propeller stirrer, and heated up to 50 °C. Then, applying constant mixing (250 RPM), the suspension stabilizers, i.e. poly(vinyl alcohol) (PVAl), and CaCl_2_ were introduced into the reactor and dissolved in water. Third, the organic phase was added to the water phase to set the suspension polymerization of VBC and DVB. The so-prepared suspension was gradually heated up to 90 °C within 4 h, and then was kept in the reactor for another 20 h upon constant mixing^38^. Afterwards, the resultant expanded-gel VBC-co-DVB copolymer was separated from reaction mixture, washed with acetone, extracted in toluene, and dried.

Next, the VBC-co-DVB copolymer was swollen in dimethyl formamide (10 mL per 1 g of the polymer), after which HEP, BAPP, and CIM were added. Next, the modification was carried out in the ERTEC 02-02 microwave reactor (Wroclaw, Poland) within a time of 15 min at the microwave radiation power of 5 W (Fig. 2a). Afterwards, the so-obtained anion exchange resins were washed with water and then successively washed with 0.1 mol L^−1^ NaOH and HCl, after which the samples were washed with 0.001 mol L^−1^ HCl. The resin (0.1 g) in hydrochloride form was introduced into 20 mL of 1000 mg Re L^−1^ solution in 0.1 mol L^−1^ HCl for 48 h. This made the ReO_4_^−^ anion exchange occur. Next, the polymer was separated by filtration, washed with redistilled water and introduced into 20 mL of water. This enabled the reduction of ReO_4_^−^ to Re^0^ via the transfer of an electron between the free electron pair located on the N atoms and the Re oxoanion. This occurred with simultaneous autooxidation of water molecules, which provided additional electrons for the process (Fig. 2b)^28,30^. The pNCs with HEP, BAPP and CIM functionalities loaded with the so-obtained ReNSs were coded Re@HEP, Re@BAPP, and Re@CIM, respectively. The samples of the pNCs were kept in their swollen form in water, and later used for further procedures.

### Catalytic reactions

For the evaluation of catalytic activity of the Re@HEP, Re@BAPP, and Re@CIM pNCs was evaluated by carrying out the reduction of 4-NP. This model reaction was selected because it proceeds in a way leading to the formation of one product, i.e. 4-aminophenol (4-AP), and does not occur without a catalyst^9^. Based on previous studies, in the present setup, the mechanism of the 4-NP reduction involves adsorption of 4-NP within the polymeric matrix that reveals affinity towards thereof^9,30^. Then, the ReNSs loaded into this polymeric matrix may react with borohydride anions to form temporary Re‒H bonds, that participate in the hydrogenation of 4-NP to 4-AP. Then, the resultant 4-AP is released from the pNCs^9,30^.

The catalytic reaction was carried out in the following way. First, 3 mL of the NAR (0.1 mmol L^−1^) was introduced into a quartz cuvette to which 0.3 mL of NaBH_4_ (0.1 mol L^−1^) was added. Next, 5–50 mg of pNC NCat (Re@HEP, Re@BAPP or Re@CIM) was introduced into the cuvette. This enabled the catalytic reduction of the 4-NP to 4-aminophenol (4-AP) at pH 9. The reduction of the 4-NP was evaluated using UV–Vis at a specified λ_max_ of 400 nm.

Based on the rate of absorbance decrease at λ_max_ 400 nm (for 4-NP), the kinetics of the catalytic processes was assessed. Because the experiment was designed in a way that involved the application of a large excess of NaBH_4_, it was possible to enable modelling using pseudo-first order kinetics. This was therefore done by plotting the *lnA*_*t*_*/A*_*0*_ vs.* t* curves, where *A*_*0*_, and *A*_*t*_ are the initial absorbance and absorbance at time *t* (s). Then, the pseudo-first order kinetics rate constant *k*_*1*_ (s^−1^) was taken from the slope of the *lnA*_*t*_*/A*_*0*_ vs.* t* plots and re-calculated to the mass-normalized rate constant *k*_*1m*_ (s^−1^ mg^−1^). Additionally, the lifetime and mass-dependent activity pf the catalysts were evaluated by calculating turnover frequency (TOF) using the following equation^46^:

1 $$TOF={n}_{4-NP}\cdot r\cdot {{n}_{Re}}^{-1}\cdot {t}^{-1}$$

where *n*_*4-NP*_ and *n*_*Re*_ are moles of 4-NP and ReNSs, respectively, *t* is the process time (min), and *r* is the yield (%) of reduction.

## Supplementary Information

Supplementary Figures.

## Data Availability

All data generated or analysed during this study are included in this published article (and its Supplementary Information files).
