# Supplementary material for: Heterogenous nanocomposite catalysts with rhenium nanostructures for the catalytic reduction of 4-nitrophenol
Source: Sci Rep. 2022 Apr 14;12:6228. doi: 10.1038/s41598-022-10237-5 (PMC9010420; doi:10.1038/s41598-022-10237-5)
Supplement: Supplementary file 1 — Supplementary Figures. [file 41598_2022_10237_MOESM1_ESM.docx]

**Supplementary Materials**

**Heterogenous nanocomposite catalysts with rhenium nanostructures for catalytic reduction of nitroaromatic compounds**

Piotr Cyganowski^1^*, Anna Dzimitrowicz^2^

^1^ Department of Process Engineering and Technology of Polymer and Carbon Materials

^2^ Department of Analytical Chemistry and Chemical Metallurgy

Faculty of Chemistry, Wroclaw University of Science and Technology

Wyb. S. Wyspianskiego 27, 50-370 Wrocław

Correspondence to: P. Cyganowski, e-mail: Piotr.cyganowski@pwr.edu.pl, phone: 71 320 23 83

ORCID: 0000-0002-3110-4246

**
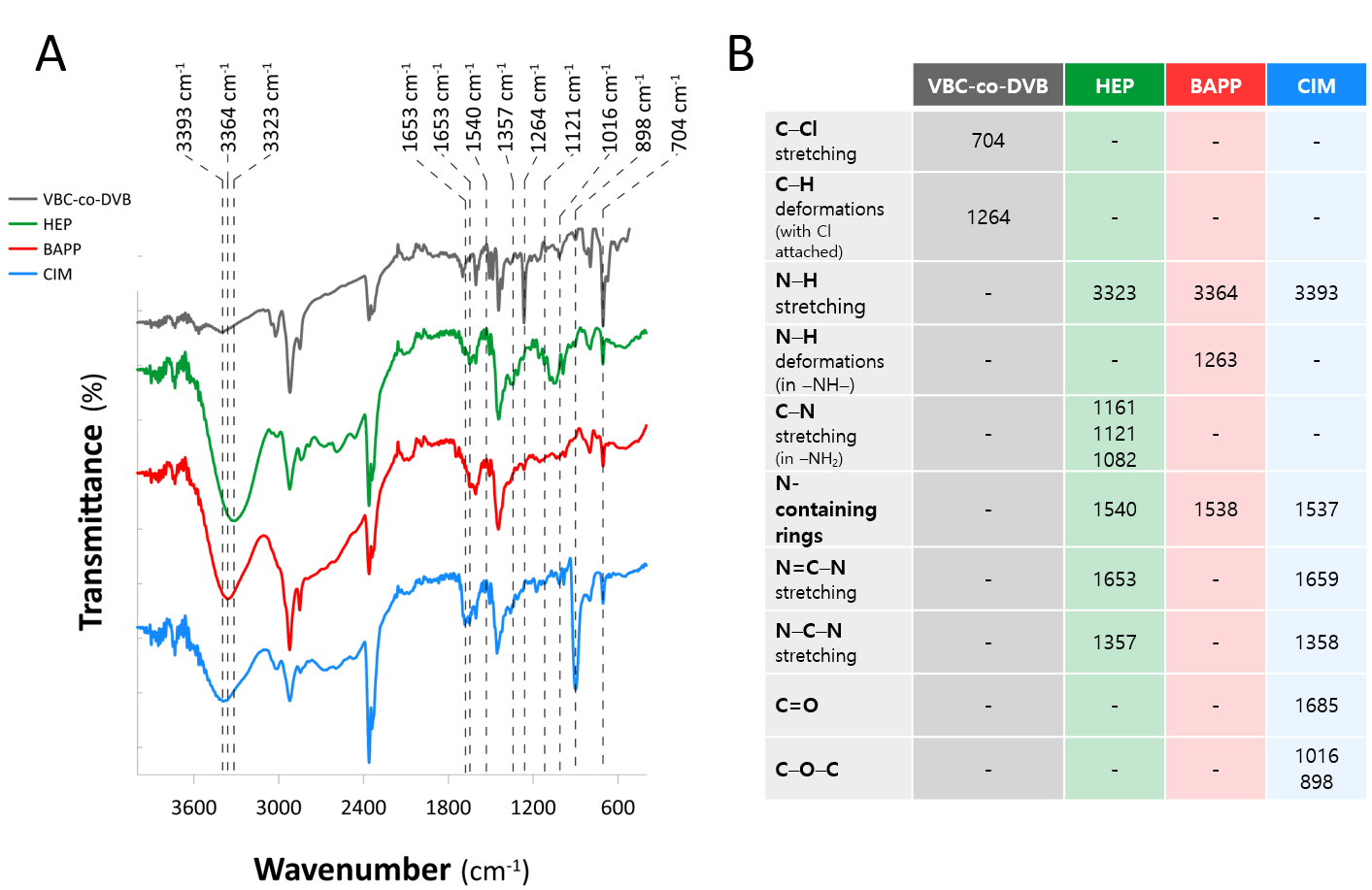
**

**Figure S1.** (**A**) ATR FT-IR spectra and (**B**) band locations of VBC-co-DVB copolymer and resins with HEP BAPP and CIM Functionalities


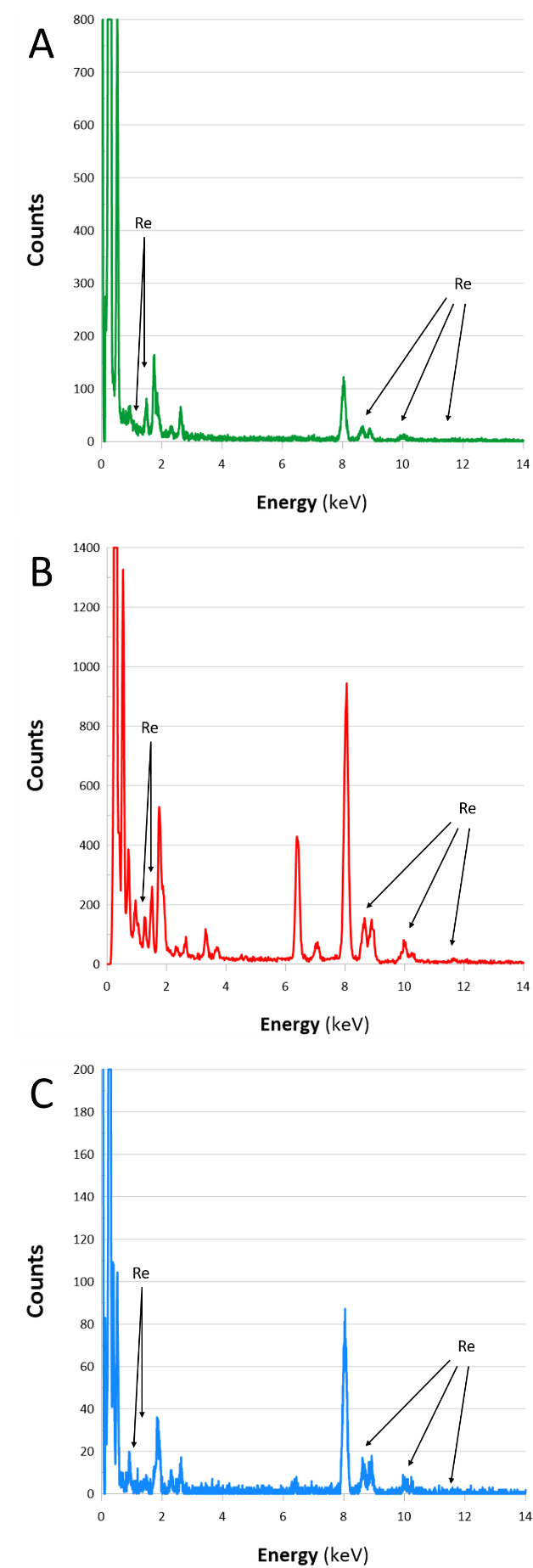


**Figure S2.** TEM/EDX spectra of nanostructures found in the (**A**) Re@HEP, (**B**) Re@BAPP, and (**C**) Re@CIM


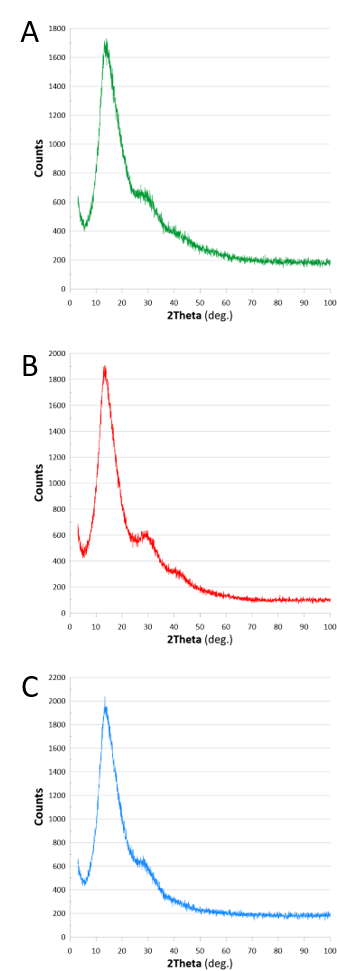


**Figure S3.** XRD spectra of (**A**) Re@HEP, (**B**) Re@BAPP and (**C**) Re@CIM


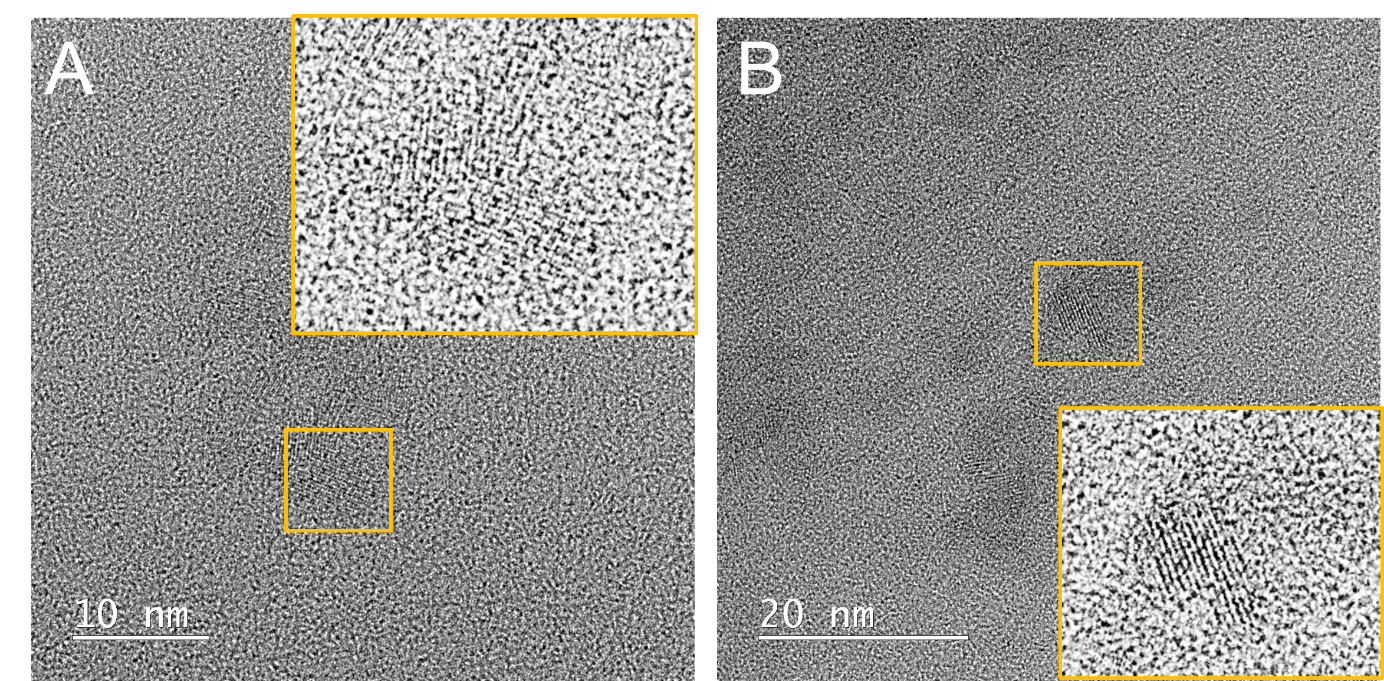


**Figure S4.** HRTEM photomicrographs used for calculating d-spacings; (**A**) Re@HEP, (**B**) Re@CIM


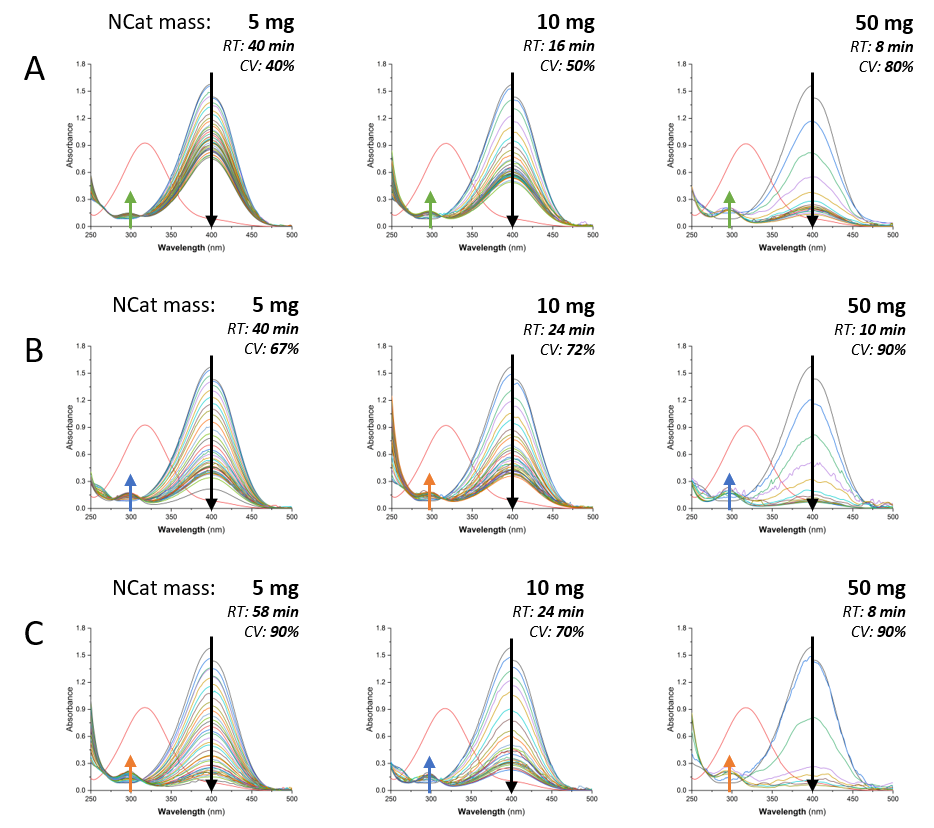


**Figure S5.** UV-Vis spectra of 4-NP reduction of 5, 10 and 50 mg of NCats. (**A**) Re@HEP, (**B**) Re@BAPP, and (**C**) Re@CIM.

**RT** and **CV** are reaction time, and 4-NP conversion taken for kinetic modelling, respectively.

Maximum reaction times were: (**A**) 105, 130, 75 min; (**B**) 119, 95, 26 min; (**C**) 98, 70, 18 min
